# Permissiveness of human hepatoma cell lines for HCV infection

**DOI:** 10.1186/1743-422X-9-30

**Published:** 2012-01-24

**Authors:** Bruno Sainz, Naina Barretto, Xuemei Yu, Peter Corcoran, Susan L Uprichard

**Affiliations:** 1 Department of Medicine, University of Illinois at Chicago, Chicago, IL 60612, USA; 2 Department of Microbiology and Immunology, University of Illinois at Chicago, Chicago, IL 60612, USA; 3 Kadmon Corporation, 450 East 29th Street, New York, NY 10016, USA; 4 Department of Medicine, Section of Hepatology, The University of Illinois at Chicago, 840 S Wood Street M/C 787, Chicago, IL 60612, USA

**Keywords:** Hepatitis C virus, Hepatoma cells, Viral permissiveness

## Abstract

**Background:**

Although primary and established human hepatoma cell lines have been evaluated for hepatitis C virus (HCV) infection *in vitro*, thus far only Huh7 cells have been found to be highly permissive for infectious HCV. Since our understanding of the HCV lifecycle would benefit from the identification of additional permissive cell lines, we assembled a panel of hepatic and non-hepatic cell lines and assessed their ability to support HCV infection. Here we show infection of the human hepatoma cell lines PLC/PRF/5 and Hep3B with cell culture-derived HCV (HCVcc), albeit to lower levels than that achieved in Huh7 cells. To better understand the reduced permissiveness of PLC and Hep3B cells for HCVcc infection, we performed studies to evaluate the ability of each cell line to support specific steps of the viral lifecycle (i.e. entry, replication, egress and spread).

**Results:**

We found that while the early events in HCV infection (i.e. entry plus replication initiation) are cumulatively equivalent or only marginally reduced in PLC and Hep3B cells, later steps of the viral life cycle such as steady-state replication, de novo virus production and/or spread are impaired to different degrees in PLC and Hep3B cultures compared to Huh7 cell cultures. Interestingly, we also observed that interferon stimulated gene (i.e. ISG56) expression was significantly and differentially up-regulated in PLC and Hep3B cells following viral infection.

**Conclusions:**

We conclude that the restrictions observed later during HCV infection in these cell lines could in part be attributed to HCV-induced innate signaling. Nevertheless, the identification of two new cell lines capable of supporting authentic HCVcc infection, even at reduced levels, expands the current repertoire of cell lines amendable for the study of HCV *in vitro *and should aid in further elucidating HCV biology and the cellular determinants that modulate HCV infection.

## Background

Worldwide, between 130 and 170 million individuals are chronically infected with hepatitis C virus (HCV), a positive-strand RNA virus that infects the liver [1,2]. Although acute infection is typically asymptomatic, ~80% of patients fail to clear the virus resulting in a chronic infection associated with the development of significant liver disease, such as fibrosis, cirrhosis, steatosis, insulin resistance and hepatocellular carcinoma (HCC) [3]. In fact, HCV-related HCC accounts for over 50% of HCC cases and over 30% of liver transplants in the United States. Despite this obvious public health burden, there is no vaccine to prevent infection and current interferon-based treatment options have toxic side effects and limited efficacy.

The main obstacle that has impeded HCV research and antiviral drug development since its discovery in 1989 [4] has been the lack of a robust infectious cell culture system capable of recapitulating all aspects of the viral lifecycle. Although early advancements in the study of HCV were made using surrogate systems [5], replicons [6-9] and HCV pseudotyped particles (HCVpp) [10], it was not until the development of the cell-culture derived HCV (HCVcc) system in 2005 that robust HCV infection was finally achieved *in vitro *[11-13]. This system was based on the identification of an HCV genotype 2a molecular clone [14], shown to be capable of replicating and assembling infectious particles in cell culture, and the discovery that the human hepatoma Huh7 cell line is permissive for HCV infection. Although numerous other human hepatoma cell lines exist, only HepG2 cells and a few other hepatoma cell lines have been rigorously tested for HCVcc permissiveness to date with varying degrees of success [11,15-20]. Identification of other cell lines able to support HCV infection would not only expand our current repertoire of cell lines available for the study of HCV, but could also prove useful for the identification of cellular determinants of HCV infection.

To identify other cell lines suitable for HCV infection studies, we assembled a panel of hepatic and non-hepatic cell lines and assessed their permissiveness for HCV infection. Here we show HCVcc infection (i.e. replication, protein translation and *de novo *virion production) in human hepatoma cell lines PLC/PRF/5 and Hep3B. Like Huh7 cells, PLC cells, a human hepatoma cell line first isolated in the early 1970s [21] and Hep3B cells, a human hepatoma cell line derived from a hepatocellular carcinoma isolated from an 8 year old male [22], have been previously utilized for HCV entry (HCVpp) [10,23-31] studies; however, to our knowledge, HCVcc infection and RNA replication in these two cell lines in the absence of complementation has not been previously reported.

## Results

### Differences in permissiveness for HCVpp and HCVcc among human hepatic and non-hepatic cell lines

Although numerous groups have assessed the permissiveness of multiple cell lines for HCVpp infection [25,32], since the development of the HCV infectious cell culture system [11-13] a comprehensive analysis of the permissiveness of human hepatic and non-hepatic cell lines for HCVcc infection has yet to be reported, and the identification of alternate cell lines that support robust HCVcc infection is still warranted. Thus, we compiled a panel of human cell lines of hepatic and non-hepatic origin (Table 1) that have been routinely used for HCVpp entry [10,25,32,33] and assessed their permissiveness for HCVcc infection.

**Table 1:** Summary of Cell Lines

Cell Line	Cell type
Huh7	Human hepatoma
HepG2	Human hepatoma
PLC/PRF/5	Human hepatoma
Hep3B	Human hepatoma
Caco-2	Human epithelial colorectal adenocarcinoma
293T	Human embryonic kidney epithelial
HeLa	Human epithelial carcinoma
SW13	Human adrenocortical carcinoma
CHO-K1	Chinese hamster ovary

**Figure 1:**
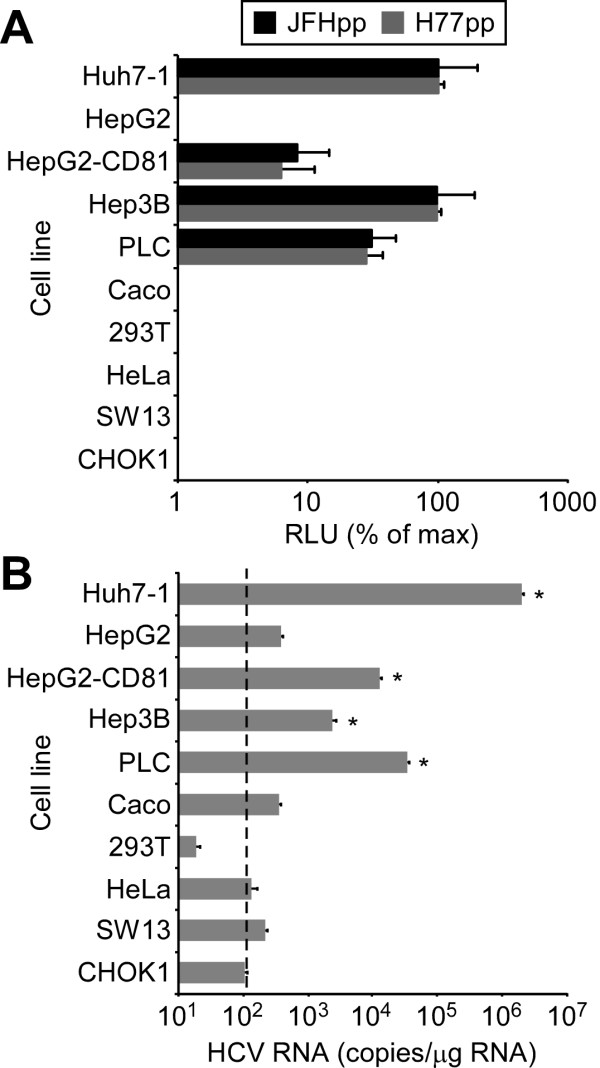
**Permissiveness of cell lines for HCVpp and HCVcc infection**. (**A**) Indicated cell lines were infected with equal amounts of JFHpp, H77pp or VSVGpp. HCVpp entry (relative light units (RLU) ± SD for triplicate samples determined 72 h p.i.) relative to Huh7-1 cells was calculated by subtracting cell line specific background and mock-control signals and then normalizing for cell line-specific VSVGpp entry levels. Results are graphed as a percentage of entry achieved in Huh7-1 infected cultures. (**B**) Indicated cell lines were infected with HCVcc at an MOI of 2.0 FFU/cell. Intracellular RNA was collected 72 h p.i. and HCV RNA was quantified by RTqPCR, normalized to GAPDH and is displayed as HCV RNA copies/μg total cellular RNA (means ± SD for triplicate samples). Significant increases in HCV RNA levels compared to CHO cells (one-way ANOVA and Tukey's post hoc *t *test) are denoted as * *p *value < 0.001. Dashed line represents background levels of non-specific bound HCV RNA.

First, for control HCVpp infections, cells were infected with pseudotyped lentiviruses encoding a luciferase reporter and bearing the glycoproteins E1 and E2 of HCV genotype 2a (JFHpp), HCV E1 and E2 of genotype 1a (H77pp) or the VSV G glycoprotein (VSVGpp). At 72 h post infection (p.i.) pseudoparticle entry was assessed by luciferase activity as previously described [34]. All cell lines were equally permissive for VSVGpp infection (8 × 10^5^-9 × 10^5 ^RLU, data not shown) however, only Huh7, Hep3B, PLC and HepG2 cells stably expressing CD81 (HepG2-CD81, Additional file 1: Figure S1) were permissive for HCVpp infection (Figure 1A). All other cell lines tested exhibited less than 1% of maximum luciferase activity, indicating that these cells are non-permissive for HCVpp entry, which is consistent with previously published reports [10].

To assess permissiveness for HCVcc infection, all cells were infected with HCVcc at an MOI of 2.0 FFU/cell and intracellular HCV RNA was determined by RTqPCR analysis 72 h p.i. We included Chinese hamster ovary (CHO-K1) cells as they lack all known HCV entry receptors and therefore serve as a control for background HCV RNA levels (i.e. non-specific cell bound HCV) (Figure 1B, dashed line = 10^2 ^copies/μg RNA) [32]. As expected, Huh7 cells were highly permissive for HCVcc and replicated HCV RNA to levels of 2.0 × 10^6 ^± 8.2 × 10^4 ^copies/*μ*g RNA by 72 h p.i. Caco-2 cell showed minimal to no HCVcc permissiveness, reaching levels of only 4.0 × 10^7 ^± 7.1 × 10^1 ^copies/*μ*g RNA. Although Caco-2 cells have previously been reported to support suboptimal HCVcc infection [35], the low levels of HCV RNA (p value > 0.05) measured in these infected cultures are likely due to receptor-bound HCV similar to that of the non-permissive parental HepG2 cell line. Addition of CD81 to the HepG2 cells conferred HCVcc permissiveness, as previously reported [11], resulting in increased HCV RNA replication of 1.3 × 10^4 ^± 4.8 × 10^2 ^copies/*μ*g RNA (*p *value < 0.01). Notably, statistically significant HCVcc infection was also detected in PLC cells and Hep3B cells. Although PLC cells were less permissive for HCVpp (Figure 1A), PLC cells contained 10-fold higher HCV RNA levels (3.9 × 10^4 ^± 2.6 × 10^3 ^copies/*μ*g RNA) 72 h after infection with HCVcc compared to Hep3B cells (2.8 × 10^3 ^± 3.5 × 10^2 ^copies/*μ*g RNA) (Figure 1B), which were as permissive as Huh7 cells for HCVpp infection (Figure 1A). Taken together, these data indicate that PLC and Hep3B cells are permissive for both HCVpp and HCVcc infection, although their permissiveness for HCVcc and HCVpp infection is reduced to different degrees compared to Huh7 cells, suggesting that PLC and Hep3B cells may differ in their capacity to efficiently support specific aspects of the HCV lifecycle. Thus, we initiated experiments to systematically dissect PLC and Hep3B permissiveness for HCVcc infection, focusing separately on each step in the viral lifecycle.

### PLC and Hep3B cells express all four HCV entry factors and support HCVcc entry

**Figure 2:**
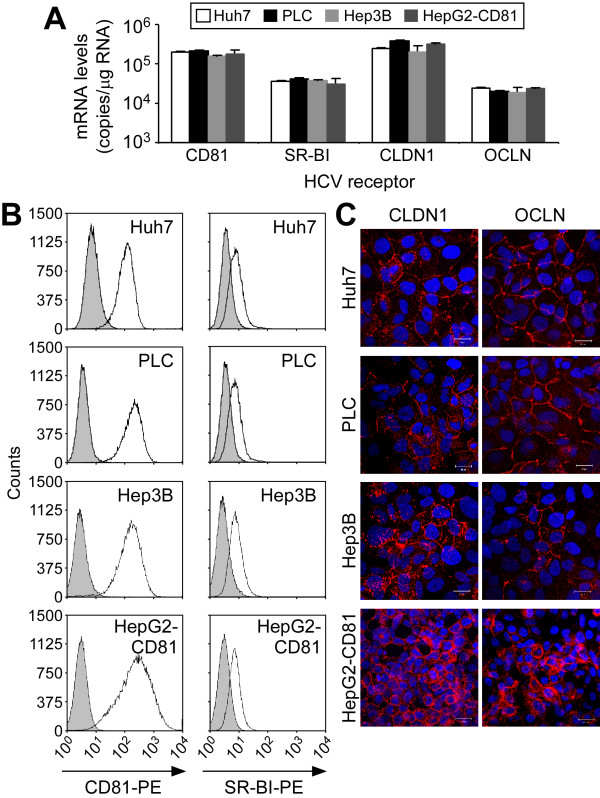
**Expression of HCV entry factors in hepatoma cells lines**. (**A**) Total RNA was extracted from Huh7, PLC, Hep3B and HepG2-CD81 cells and mRNA copies were determined by RTqPCR using standard curves comprised of DNA plasmids expressing the coding sequence of the gene of interest. Absolute quantities were normalized to GAPDH and data are displayed as mRNA copies/μg total cellular RNA (means ± SD). (**B-C**) Cell surface expression of HCV entry factors on Huh7, PLC, Hep3B and HepG2-CD81 cells was determined by flow cytometric analysis or IF. (**B**) For flow cytometric analysis, cells were trypsinized and stained with mouse anti-CD81 or anti-SR-BI monoclonal antibodies and an anti-mouse secondary antibody conjugated with PE. Shaded regions represent cells stained with a monoclonal mouse IgG control primary antibody and an anti-mouse PE-conjugated secondary antibody. (**C**) For IF analysis, fixed cells were stained with antibodies specific for CLDN1 or OCLN and counterstained with a species specific Alexa-555-conjugated secondary antibody. Indicated protein is red (Alexa 555) and nuclei are blue (Hoechst). Magnification × 630; scale bar = 20 μm.

PLC cells [21], Hep3B cells [22] and HepG2-CD81 cells are hepatoma-derived cells lines which although differ cytologically from one another share similar hepatocytes-like characteristics with Huh7 cells, such as cuboidal epithelial-like morphology with high nucleus-to-cytoplasm ratio, containing mono- and bi-nuclei with multiple nucleoli and cytoplasmic granules (Additional file 1: Figure S2). Our initial screening (Figure 1) indicated that these three cells lines also share permissiveness for both HCVpp and HCVcc infection albeit to differing degrees. Since viral binding/entry represents the first step in the HCV lifecycle, we first assessed the expression of the known HCV entry factors in these cells by measuring CD81, SR-BI, CLDN1 and OCLN mRNA levels by RTqPCR analysis (Figure 2A). When compared to Huh7, no significant difference in mRNA expression for the four known HCV entry factors was observed among the four cell lines. We next analyzed the cell surface expression of CD81 and SR-BI by flow cytometry and CLDN1 and OCLN by indirect immunofluorescence (IF) as our commercially-available antibodies to these two tight junctions proteins are not amendable to flow cytometric analysis. As shown in Figure 2B, cell surface expression of CD81 and SR-BI in PLC, Hep3B and HepG2-CD81 cells, as measured by flow cytometry, was comparable to that observed on Huh7 cells. IF analysis of CLDN1 and OCLN also confirmed cell surface expression of these receptors on the newly identified HCVcc-permissive PLC and Hep3B cells as well as the previously characterized Huh7 [36] and HepG2-CD81 cells [35]. Taken together, these data indicate that PLC and Hep3B cells, at a minimum, express and localize all four known HCV entry receptors on their cell surface.

**Figure 3:**
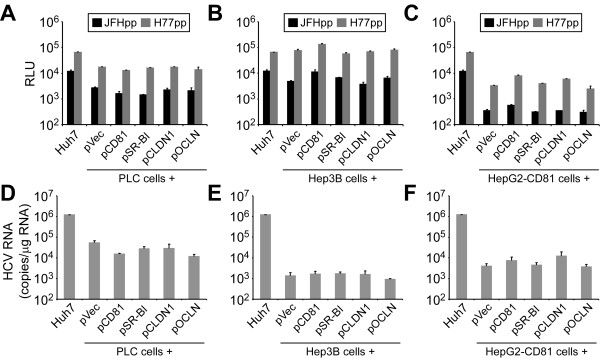
**HCVpp and HCVcc infection in PLC, Hep3B and HepG2-CD81 cells exogenously over expressing HCV entry factors**. Huh7, PLC, Hep3B and HepG2-CD81 cells transiently transfected with a vector control plasmid or expression plasmids expressing CD81, SR-BI, CLDN1 and/or OCLN were infected with (**A-C**) equal amounts of JFHpp, H77pp or VSVGpp or (**D-F**) HCVcc at an MOI of 2.0 FFU/cell. HCVpp entry (following background and mock-control signal subtraction) is expressed as relative light units (RLU) ± SD for triplicate samples determined 72 h p.i. Intracellular HCVcc RNA was quantified by RTqPCR, normalized to GAPDH and is displayed as mean HCV RNA copies/μg total cellular RNA ± SD for triplicate samples determined 72 h p.i.

Despite expression all four of the previously established essential HCV entry factors, the PLC and HepG2-CD81 cells showed a reduced permissiveness for HCVpp infection (Figure 1A) when compared to Huh7 and Hep3B cells. While varying luciferase activity after parallel HCVpp inoculation is a phenotype we have seen among Huh7 cell lines from different laboratories (Additional file 1: Figure S3 and [34]), to functionally test whether these cells express suboptimal levels of any of the known HCV entry factors, we transiently transfected PLC, Hep3B and HepG2-CD81 cells with vectors expressing the four known human HCV receptors and reassessed their permissiveness for HCVpp and HCVcc infection 48 h post-transfection. As shown, expression of HCV entry factors *in trans *individually (Figure 3) or in combination (data not shown) did not enhance HCVpp (Figure 3A-C) or HCVcc (Figure 3D-F) infectivity of PLC, Hep3B or HepG2-CD81 cells. Hence, the 65% less luciferase activity generated when PLC cells were inoculated with HCVpp compared to Huh7 and Hep3B cells, is not due to suboptimal expression of any of the 4 known HCV entry factors.

### PLC and Hep3B cells can support equivalent levels of HCV RNA replication as Huh7 cells

**Figure 4:**
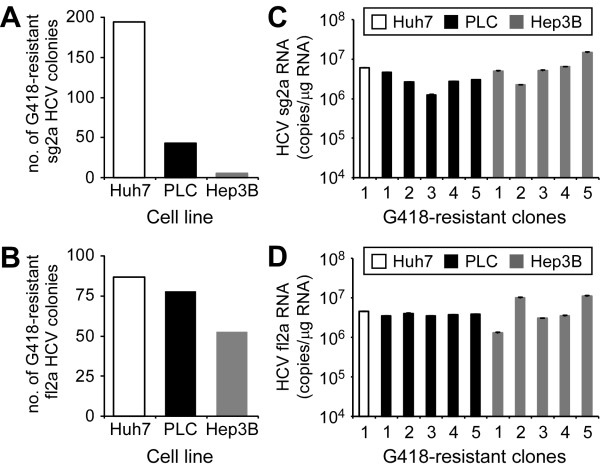
**HCV sub-genomic and full-length replicon RNA replication in PLC and Hep3B cells**. (**A**) Huh7, PLC and Hep3B cells were transfected with 1 μg of *in vitro*-transcribed sg JFH-1 RNA via electroporation. Cells were diluted 1:500 and plated in 100 mm tissue culture dishes and maintained in the presence of 500 μg/ml G418 for 2 weeks. Colonies were fixed, stained with crystal violet and counted. (**B**) Huh7, PLC and Hep3B cells were infected with 100 FFU of HCVcc^*G418 *^and maintained in the presence of 500 μg/ml G418 for 2 weeks. Colonies were fixed, stained with crystal violet and counted. (**C-D**) HCV G418-resistant sg and fl replicon clones were expanded in the presence of 500 μg/ml G418 and total RNA extracted ~3 weeks post expansion. HCV RNA was quantified by RTqPCR, normalized to GAPDH and is displayed as mean HCV RNA copies/μg total cellular RNA ± SD for each clone.

Defects in post entry steps, such as reduced HCV RNA replication, can negatively affect HCVcc infectivity even if entry is achieved. Since both PLC and Hep3B cells appear to be entry competent to reasonable degrees, their more significantly reduced permissiveness for HCVcc infection compared to Huh7 cells may be due to defects in HCV RNA replication. To determine their capacity to support HCV RNA replication, PLC and Hep3B cells were transfected with HCV genotype 2a subgenomic (sg2a) replicon *in vitro*-transcribed RNA, and G418-resistant colony formation was determined as has been previously described for Huh7 [37] and HepG2 cells [18]. HCV sg2a replicons could be readily established in both cell lines; however, the ratio of colony formation to μg of *in vitro*-transcribed RNA was considerably lower in both Hep3B and PLC cells compared to Huh7 cells (Figure 4A). Since this reduction could be attributed to differences in transfection efficiency, we introduced HCV by an alternative means in which PLC and Hep3B cells were infected with medium from Huh7 cells replicating the full length 2a (fl2a) HCV replicon that contains infectious HCVcc capable of conferring G418 resistance to newly infected cells if entry is achieved and replication is established [38]. Unlike the results obtained following transfection of PLC and Hep3B cells with sg2a RNA, the number of G418-resistant colonies established in both PLC and Hep3B cells infected with HCVcc^*G418 *^was more comparable to that achieved in Huh7 cells (Figure 4B), although a 65% reduction in Hep3B colony formation was noted (*p *= 0.02). We next expanded some of these G418-resistant sg2a and fl2a PLC and Hep3B clones and quantified HCV RNA levels in each clone to determine steady-state HCV RNA replication levels achieved in each cell line. RTqPCR analysis revealed relatively similar levels of HCV sg2a RNA replication in all three cell lines, with no significant difference (*p *value > 0.05) in mean HCV RNA copies/μg intracellular RNA observed between Huh7, PLC and Hep3B G418-resistant sg2a clones (Figure 4C; 5.9 × 10^6 ^± 7.2 × 10^3 ^vs. 3.9 × 10^6 ^± 6.0 × 10^5 ^and 6.7 × 10^6 ^± 2.4 × 10^6^, respectively). Likewise, no significant difference (p value > 0.05) in the levels of intracellular HCV RNA in fl2a HCVcc^*G418*^-established clones was observed between Huh7, PLC and Hep3B G418-resistant fl2a clones (Figure 4D; 4.5 × 10^6 ^± 1.8 × 10^4 ^vs. 3.9 × 10^6 ^± 6.7 × 10^4 ^and 5.8 × 10^6 ^± 1.4 × 10^6 ^HCV RNA copies/*μ*g, respectively). Importantly, since G418-resistant colony formation following HCVcc^*G418 *^infection is dependent on both successful viral entry and efficient HCV RNA replication, the fact that similar numbers of G418-resistant colonies and equal levels of HCV RNA replication were achieved in both PLC and Hep3B, compared to Huh7 cells, would indicate that despite individual differences in the efficiency of HCV entry or HCV replication between these cell lines together the cumulative efficiency of HCVcc entry plus subsequent RNA replication is relatively equivalent in PLC, Hep3B, and Huh7 cells, thus allowing equivalent stable infection initiation events to occur (i.e. fl2a colony formation). However, to rule out the possibility that HCVcc^*G418 *^infection could have selected for a population of PLC or Hep3B cells with enhanced efficiency for viral entry and/or replication, we cured fl2a PLC and Hep3B replicon clones by IFN-treatment and re-assessed their permissiveness for HCVcc infection. As shown in Additional file 1: Figure S4, none of the IFN-cured fl2a clones showed enhanced permissiveness for HCVcc infection compared to their parental control.

### PLC, Hep3B and HepG2-CD81 cells are defective for de novo HCVcc production

**Figure 5:**
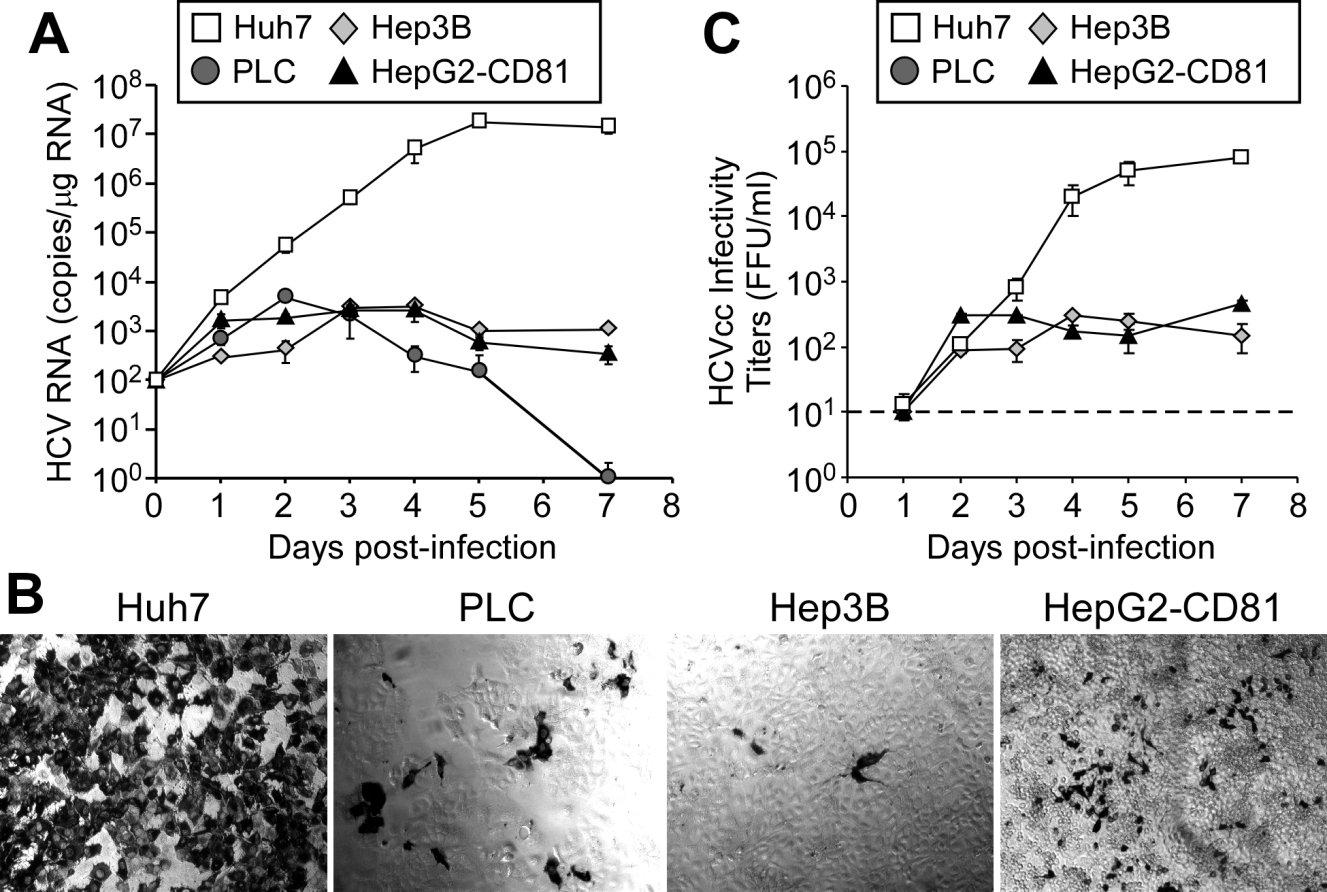
**HCVcc infection kinetics in hepatoma cells**. Huh7, PLC, Hep3B and HepG2-CD81 cells were infected with JFH-1 HCVcc at an MOI of 0.01 FFU/cell. Culture supernatant and intracellular RNA were collected at the indicated times p.i. (**A**) Intracellular HCV RNA was analyzed by RT-qPCR, normalized to GAPDH and is displayed as HCV copies/μg total RNA (means ± SD for triplicate samples). (**C**). On day 5 p.i., parallel cultures of infected cells were fixed and stained for HCV E2 [39] (magnification, 100×). (**C**) Infectivity titers, expressed as FFU/ml, were determined on naïve Huh7-1 cells. The dashed line represents the lower limit of detection of the assay (10^1 ^FFU/ml).

Thus far, our results indicate that cumulatively the early events in HCV infection (i.e. entry plus replication initiation) are equivalent or only marginally reduced in PLC and Hep3B, respectively compared to Huh7 cells. Therefore, the 1.5 and 3 log reduction in HCVcc permissiveness observed for the PLC and Hep3B cell lines, respectively (Figure 1A), is likely attributable to a defect(s) in a post replication step, such as assembly and/or egress of *de novo *HCVcc. To monitor multiple rounds of HCV infection and spread, which is dependent on HCV assembly and egress, we performed a kinetic analysis of HCV infection over the course of 7 days in Huh7, PLC, Hep3B and HepG2-CD81 cells after infection with HCVcc at a low MOI (Figure 5). Using this approach, we show that intracellular HCV RNA levels (Figure 5A) in Huh7 cells infected with HCVcc at an MOI of 0.1 FFU/cell reached 4.44 × 10^3 ^± 4.84 × 10^2 ^copies/*μ*g RNA by one day p.i. and increased exponentially thereafter ~3,000-fold reaching a steady-state level of 1.6 × 10^7 ^± 2.0 × 10^6 ^copies/*μ*g RNA by day 5 p.i. HCV-infected PLC cells showed parallel but slightly reduced HCV RNA expansion kinetics during the first 48 h p.i. compared to HCV-infected Huh7 cells; however, by day 3 p.i., HCV RNA levels began to decline reaching undetectable levels by day 7 p.i. In HCV-infected Hep3B cells, following a 1-2 log increase in HCV RNA levels during the first 72 h p.i., HCV RNA reached and maintained a low steady-state level of ~2.1 × 10^3 ^± 6.9 × 10^2 ^copies/*μ*g RNA. Similarly HepG2-CD81 cells clearly lacked a notable robust expansion in HCV RNA levels after 24 h p.i. and maintained a low steady-state level (1.6 × 10^3 ^± 7.1 × 10^2 ^copies/*μ*g RNA) until day 7 p.i. Likewise, when entry was bypassed by transfecting cells with infectious full-length HCV JFH-1 *in vitro*-transcribed RNA, a similar profile of RNA expansion and kinetics was observed (Additional file 1: Figure S5).

Analysis of HCV-positive cells by immunocytochemical staining of HCV E2 protein 5 days p.i (Figure 5B), indicated that while the majority of cells in the Huh7 culture had become infected, only a small percentage of the cells in the PLC, Hep3B and HepG2-CD81 cultures were infected likely representing the 10% of cells that where initially infected by the viral inoculum. Since viral spread to a large extent is mediated by *de novo *secretion of infectious HCV virions, we next quantified the level of infectious HCVcc secreted into the supernatants of each infected cell line over the course of 7 days (Figure 5C) using a standard HCV foci formation assay as described [40]. While infectious HCVcc was detectable in Hep3B and HepG2-CD81-infected cultures at reduced levels, infectious HCVcc was undetectable (i.e. below the lower limit of the assay) in the culture supernatant of HCV-infected PLC cells even at day 2 when the PLC cultures had amplified a higher level of intracellular HCV RNA than the Hep3B and HepG2-CD81 cells (Figure 5C).

### PLC cells exhibit reduced HCV RNA encapsidation and HCVcc egress

**Figure 6:**
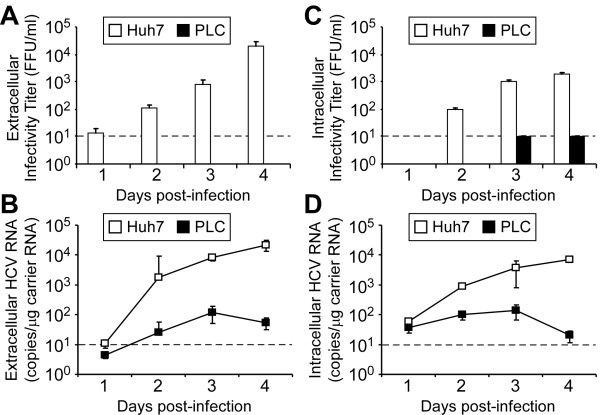
**HCV extracellular and intracellular infectivity titers and RNA genome copies in Huh7 and PLC cells**. Huh7 and PLC cells were infected with JFH-1 HCVcc at an MOI of 1.0 FFU/cell. Culture supernatant and cells were collected at the indicated times p.i. (**A**) Extracellular infectivity titers, expressed as FFU/ml, were determined on naïve Huh7-1 cells. The dashed line represents the lower limit of detection of the assay (10^1 ^FFU/ml). (**B**) For extracellular RNA analysis, culture supernatant samples were treated with S7 Micrococcal nuclease prior to RNA purification. Two μg of total murine carrier RNA was added to all samples for normalization. HCV RNA values were determined by RT-qPCR and are displayed as HCV copies/μg total carrier RNA (means ± SD for triplicate samples). The dashed line represents the background of the assay (10^1 ^HCV RNA copies/μg carrier RNA). (**C-D**) For intracellular infectivity titer and RNA analysis, cells were pelleted, re-suspended in complete 10% cDMEM and lysed via multiple freeze/thaws. (**C**) Intracellular infectivity titers, expressed as FFU/ml, were determined on naïve Huh7-1 cells. The dashed line represents the lower limit of detection of the assay (10^1 ^FFU/ml). (**D**) Intracellular HCV RNA copies were determined after S7 Micrococcal nuclease treatment by RT-qPCR and are displayed as HCV copies/μg total carrier RNA (means ± SD for triplicate samples). The dashed line represents the background of the assay (10^1 ^HCV RNA copies/μg carrier RNA).

To distinguish whether the lack of infectious HCVcc present in the supernatant of HCV-infected PLC cells (Figure 5C) is due to a defect in *i) *intracellular HCV RNA encapsidation and virion assembly and/or *ii) *release of *de novo *infectious HCVcc, we infected Huh7 and PLC cells with HCVcc at an MOI of 1.0 FFU, collected cells and culture medium from infected cultures over the course of 4 days, and quantified intracellular and extracellular HCVcc infectivity titers and viral RNA. Consistent with the data shown in the 7 day low MOI infection experiment (Figure 5C), increasing infectious HCVcc levels were detected in the supernatant of HCV-infected Huh7 cells; however, at no time p.i. did we detect infectious HCVcc in the culture supernatants from HCV-infected PLC cells (Figure 6A). To determine whether non-infectious HCVcc virions were secreted from the infected PLC cells, supernatants were treated with S7 Micrococcal nuclease, RNA extracted and HCV genome copies quantified by RTqPCR. As shown in Figure 6B, extracellular nuclease-protected HCV RNA (i.e. encapsidated viral RNA) was detected in culture supernatant harvested from HCV-infected Huh7 cells, and levels exponentially increased over the course of infection, consistent with the production and excretion of *de novo *infectious HCVcc. In HCV-infected PLC cells, however, significantly lower levels of nuclease-protected HCV RNA were detected with a modest exponential increase in total copies observed between days 1 and 3. Notably, if the HCV RNA present in the PLC cell supernatant did reflect infectious HCVcc, the level of infectivity (~ 1-2 FFU/ml) would be below the level of detection in our titration assay (10 FFU/ml). Thus, we can not conclude from this whether the low level of HCV RNA secreted by PLC cells represents infectious or non-infectious virions. To assess the presence and infectivity of intracellular HCV virions, cells were washed extensively with 1 × PBS, trypsinized, resuspended in 10% cDMEM, pelleted at 1,400 RPM for 5 min and resuspended cell pellets subjected to multiple freeze thaws. Cell debris was removed by centrifugation and the presence of infectious HCVcc in cleared lysates was determined by foci titration analysis and the presence of non-infectious HCV by RNA analysis following S7 Micrococcal nuclease treatment. As expected, increasing levels of infectious HCVcc and nuclease-protected HCV RNA were detected in intracellular lysates from HCV-infected Huh7 cells over the course of infection (Figure 6C and 6D, respectively). In HCV-infected PLC cells, low levels of infectious HCVcc were detectable only on days 3 and 4 p.i. (Figure 6C), and similar to the levels of extracellular HCV RNA detected in culture supernatants (Figure 6B), low levels of nuclease-protected HCV RNA were detected in intracellular lysates, again with a modest exponential increase in total copies observed between days 1 and 3. Together, these data indicate that the ability of PLC cells to efficiently encapsidate HCV RNA is markedly reduced compared to Huh7 cells, resulting in low to undetectable levels of secreted infectious *de novo *HCVcc.

### Non-Huh7 hepatoma cells induce the expression of IFN-stimulated genes following HCVcc infection

**Figure 7:**
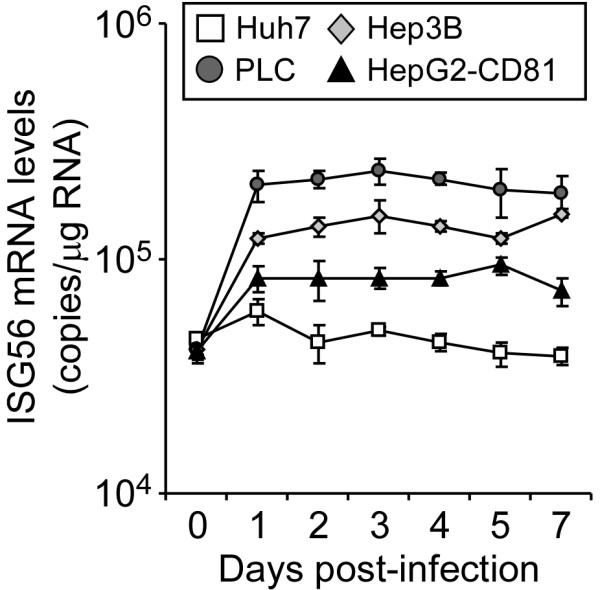
**ISG56 mRNA induction in HCVcc-infected hepatoma cells**. Huh7, PLC, Hep3B and HepG2-CD81 cells were infected with JFH-1 HCVcc at an MOI of 1.0 FFU/cell and intracellular RNA was collected at the indicated times p.i. (**A**) Intracellular ISG56 mRNA was analyzed by RT-qPCR, normalized to GAPDH and is displayed as ISG56 mRNA copies/μg total RNA (means ± SD for triplicate samples).

Viruses often induce a cellular innate immune response, mediated by the induction of IFN-stimulated genes (ISG), in the infected host cell [41]. Although the cellular ISGs and pathways induced by viral infections are generally similar among mammalian cells (reviewed in [42]), different cell lines possess different abilities to mount specific antiviral responses to the same virus, which can potentially cause differences in permissiveness to viral infections. This has recently been demonstrated by Zhao *et al*., for murine coronavirus infection of different murine cell lines [43]. To determine if HCV differentially induces the expression of ISGs in Huh7, PLC, Hep3B and HepG2-CD81 cells following infection, we evaluated ISG56 mRNA induction by RTqPCR following infection of cells with HCVcc at an MOI of 1.0 FFU/cell. As shown in Figure 7, ISG56 mRNA levels were not up-regulated in HCV-infected Huh7 cells and remained constant throughout the course of infection, indicating that as previously shown [44], HCVcc does not induce an efficient innate interferon response in Huh7 cells. In contrast, induction of ISG56 was observed in the other three human hepatoma cell lines following HCVcc infection. Specifically, ISG56 mRNA levels were up-regulated 3- and 2-fold in Hep3B and HepG2-CD81 cells, respectively, as early as 24 h p.i. and reached a steady state level by 3 days post infection. The highest and most significant induction of ISG56, however, was observed in HCV-infected PLC cells. Notably, we have previously shown that IFN-mediated activation of the innate cellular interferon response inhibits HCV RNA replication and subsequent production of *de novo *HCV virions ([45] and data not shown), thus the reduced steady-state HCVcc infection levels observed in Hep3B and HepG2-CD81 cells and the decline of HCV infection observed in PLC cells, despite these cells exhibiting comparable infection initiation permissiveness, may be at least in part a consequence of HCV-induced innate immune signaling in these hepatoma cell lines.

## Discussion

In this study we characterized a panel of hepatic and non-hepatic cell lines (Table 1) for HCVcc permissiveness. All non-hepatic cell lines were either refractory or marginally permissive for HCVpp and HCVcc infection (Figure 1), as previously shown [10,25,30,32,35]; however, contrary to commonly held perception, all three of the hepatoma cell lines tested were permissive for HCVcc infection, although to varying degrees relative to Huh7 cells.

### Permissiveness of hepatoma cell lines for HCVpp and HCVcc infection

Considering the phenotypic differences observed among the hepatoma cell lines (Additional file 1: Figure S2), the perception that Huh7 cells are relatively unique in their ability to support HCV infection [34,46], and based on previous reports demonstrating varying degrees of HCVcc infection in other hepatoma cell lines, such as HepG2, HepaRG, and immortalized human hepatoma (IHH) cells [15,16,18-20,32,47], we were surprised that HCVcc infection and replication could be readily detected in both Hep3B and PLC cells (Figure 1). In fact, HCV RNA levels exceeding 10^4 ^copies/μg RNA were detected in PLC cells 3 days post infection with HCVcc. When compared directly to Huh7 cells, however, it was apparent that HCVcc infection in both PLC and Hep3B cells was reduced (2.0 × 10^6 ^± 8.2 × 10^4 ^vs. 3.9 × 10^4 ^± 2.6 × 10^3 ^and 2.8 × 10^3 ^± 3.5 × 10^2 ^copies/*μ*g RNA, respectively). In addition, our initial screen also showed notable differences in permissiveness for HCVpp infection between these two cell lines. Specifically, HCVpp infectivity was ~65% lower in PLC cells compared to HCVpp-infected Huh7 and Hep3B cells. However, although significant (*p *value = 0.03 and 0.02 for JFHpp and H77pp, respectively), we have seen similar variability in permissiveness for HCVpp infection even among fully HCVcc-permissive Huh7 cell lines from different laboratories (Additional file 1: Figure S3 and [34]), indicating that this level of HCV entry should be sufficient to support robust HCVcc infection. Consistent with this hypothesis, permissiveness for HCVcc infection initiation was notably higher in PLC cells compared to HCV-infected Hep3B cells (Figure 1B and 4B), despite the lower permissiveness of the PLCs for HCVpp entry (Figure 1A). Taken together, the results from our initial screen (Figure 1) indicated that PLC and Hep3B cells are indeed permissive for both HCVpp and HCVcc infection; however, differences in permissiveness compared to Huh7 cells and between each cell line do exist, and thus we initiated a systematic analysis of PLC and Hep3B permissiveness for HCVcc infection, performing experiments that focused on the different steps of the viral lifecycle (i.e. entry, replication, egress and spread).

i. HCV entry. To determine if the differences in HCVpp and HCVcc infection between each hepatoma cell line could be attributed to entry factors already shown to modulate HCV permissiveness in other hepatoma cell lines [25,32,48-51], we examined the expression of four essential HCV entry factors (i.e. CD81, SR-BI, CLDN1 and OCLN) by RTqPCR, flow cytometric and immunofluorescence analysis (Figure 2). Interestingly, the data indicated that when compared to the fully permissive Huh7 cell line, no obvious differences in mRNA level or cell surface expression of CD81, SR-BI, CLDN1 or OCLN was observed in PLC, Hep3B or HepG2-CD81 cells. In addition, we over-expressed each of the four HCV entry factors in each cell line since we and others have shown that expression of HCV entry factors *in trans *can enhance HCVpp and HCVcc entry in cells where HCV permissiveness is low [25,32,34]. Consistent with the observation that these cell lines already express the necessary levels of the four HCV entry factors, over-expression of CD81, SR-BI, CLDN1 or OCLN individually or in combination did not significantly increase HCVpp or HCVcc entry in any of the hepatoma cell lines (Figure 3). Hence, the current data indicate that this panel of hepatoma cell lines expresses the minimum set of known entry factors necessary for efficient HCV entry. However, we cannot rule out the possibility that the cells tested are deficient in another HCV entry factor (e.g. the transferin receptor 1 protein [52] or the Niemann-Pick C1-Like 1 cholesterol absorption receptor [53], which we recently showed to be HCV entry factors), entry cofactors (e.g. epidermal growth factor receptor and ephrin receptor A2 [54]) or a post entry host cellular protein required for efficient viral fusion or nucleocapsid uncoating.

ii. HCV RNA replication. To examine HCV replication independent of inherent differences in PLC and Hep3B cell transfection efficiency, we took advantage of the G418-selectable HCVcc^*G418 *^virus [38], which can be used to establish HCV fl2a replicon cells via direct infection of cells rather than electroporation. Using this approach, 88, 77 and 52 G418-resistant colonies were formed in Huh7, PLC and Hep3B cultures, respectively, 3 weeks following infection with 100 FFU of HCVcc^*G418 *^(Figure 4B). Additional analysis of HCV RNA replication levels in selected fl2a clones revealed that comparable levels of HCV RNA replication were achieved in all three cell lines (Figure 4D). Likewise similar levels of intracellular HCV RNA were achieved in sg2a replicon cell clones (Figure 4C). Importantly, since G418-resistant colony formation following HCVcc^*G418 *^infection is dependent on both successful viral entry and efficient HCV RNA replication, the fact that similar numbers of G418-resistant colonies and equal levels of HCV RNA replication were achieved in both PLC and Hep3B cells, compared to Huh7 cells, indicate that the cumulative efficiency of viral entry plus subsequent RNA replication initiation is relatively equivalent between PLC, Hep3B and Huh7 cells.

iii. Spread and infectious HCVcc production. By performing a 7 day low MOI infection assay, we were able to assess the efficiency of viral spread by assessing intracellular HCV RNA expansion relative to *de novo *infectious HCVcc production and by visualizing viral spread directly by immunostaining for HCV-positive cells. Analysis of HCV RNA replication following infection of each cell line with HCVcc (Figure 5A) or electroporation with full-length infectious HCV JFH-1 *in vitro*-transcribed RNA (Additional file 1:Figure S5) revealed that while intracellular HCV RNA initially increased in all the infected/transfected cell cultures at different rates, it was only in Huh7 cultures where HCV RNA levels continued to increase exponentially past day 2 or 3 post infection/transfection. Notably, exponential increase of viral RNA in these cultures after day 2-3 p.i. would require spread of the virus from the original infected/transfected cells. Consistent with this, HCV E2 staining on day 5 post-HCVcc infection revealed that the vast majority of Huh7 cells were HCV-positive while little to no spread was evident in the PLC, Hep3B or HepG2-CD81 cell cultures (Figure 5B). Thus, while infection could be initiated in all these cell lines, only Huh7 cells appeared to support efficient viral spread. Since spread of HCV to naïve cells following infection is dependent on secretion of *de novo *infectious virions, we measured infectious HCVcc in the supernatant of infected cultures and observed detectable but reduced levels of infectious HCVcc in Hep3B and HepG2-CD81 culture supernatants and undetectable levels of HCVcc in infected PLC cultures (Figure 5C). Low steady-state levels of intracellular HCV RNA and infectious virus production in the Hep3B and HepG2-CD81 cells could result from a defect/bottleneck in any phase of the viral life cycle (e.g. reduced and/or ISG inhibition of entry, replication or secretion); however, since the cumulative efficiency of viral entry plus subsequent initiation of HCV RNA replication appears to be relatively equivalent between Hep3B, HepG2-CD81 and Huh7 cells (Figure 4B), it is reasonable to speculate that robust HCV infection in Hep3B and HepG2-CD81 cells may be limited by a decreased capacity for virus production compared to Huh7 cells. Of course, in the case of PLC cells, there was a clear and severe defect in HCVcc virus production. Not only were no infectious particles detected in the supernatant of infected cultures (Figure 5B and 6A), but analysis of HCVcc present within the cell revealed low to undetectable levels of intracellular infectious virions (Figure 6C) and comparably low levels of encapsidated HCV RNA in both these extra- and intra-cellular samples (Figure 6B and 6D) suggesting virion assembly is restricted at or before genome encapsidation.

### Cellular determinants of HCV permissiveness

The reduced ability of PLC, Hep3B and HepG2-CD81 cell cultures to support robust steady-state infection levels or spread of HCVcc is likely multifactorial and could be directly due to or influenced by the lack of sufficient levels of a cellular protein necessary for efficient production of infectious progeny virus or a consequence of the presence of an inhibitory factor.

i. ISG induction. Since induction of innate immune signaling can have negative affects on multiple steps in the HCV lifecycle, we assessed the ability of PLC, Hep3B and HepG2-CD81 cells to mount an innate immune response against HCV infection by measuring ISG56 mRNA induction, as a representative ISG, following HCVcc infection. Importantly, while no induction of ISG56 was observed in Huh7 cells, as previously published [44], ISG56 mRNA was up-regulated in HCV-infected PLC, Hep3B and HepG2 cells, with PLC cells exhibiting the most significant induction following HCVcc infection (Figure 7). Although it is generally believed that most cultured hepatoma cells generally are impaired for poly(I-C)- and virus-activated IFN responses [55], these results are consistent with previous findings that IFN-β promoter activity is more potently induced in HepG2 and Hep3B cells than in Huh7 cells following either liposome-mediated transfection of poly(I-C) or Sendai virus infection [56]. Relevant to this, a more recent publication by Marukian et al. [57] shows a correlation between innate antiviral gene induction and impaired HCV replication and spread in primary liver cells. Likewise, HCV spread was not observed in highly differentiated microscale primary human hepatocyte cultures described by Ploss *et al*., (2010), who speculated that while the expression of viral proteins in the initially infected cells might be sufficient to blunt intracellular antiviral signaling and allow sustained viral replication, interferon produced by the infected cell may be sufficient to signal to/activate adjacent naïve cells rendering them resistant to infection and thus limiting subsequent viral spread [58]. Thus, the differences in ISG induction observed between these cells lines (Figure 7) may keep HCV replication lower on a per cell basis (Hep3B and HepG2-CD81) and/or at higher levels successfully inhibit viral replication in the infected cells sufficiently to contribute to clearance of the infection (PLC).

ii. miR-122. While ISGs are well known negative determinants of HCV permissiveness, numerous positive determinants required for efficient HCV replication have also been identified (reviewed in [59]). Although overlap between independent studies is not often observed, leaving the relevance of many of these factors to be confirmed, new data from multiple laboratories has recently demonstrated that human micro RNA (miR122) is a critical liver-specific factor required for robust HCV infection and secretion [60-65]. Because IFN treatment has been shown to reduce miR122 expression in Huh7 cells [66], it is possible that the enhanced ISG induction observed in the other three cell lines may more potently reduce this important positive regulator of HCV infection. Likewise, miR122 has been shown to play an important role in regulating cholesterol homeostasis, fatty acid metabolism and lipogenesis [63-65,67], three lipid metabolic pathways shown to be necessary for HCV replication, assembly and secretion. In fact, just prior to submitting this manuscript, Evans and colleagues and Matsuura and colleagues separately showed that exogenously expressing human miR122 in HepG2 or Hep3B cells, respectively, results in robust HCV replication and infectious virus secretion following HCVcc inoculation or HCV RNA transfection [19,20]. Hence, lack of adequate miR122 may also contribute to the reduced HCV permissiveness of certain cell lines.

In summary, by testing a panel of human hepatoma and non-hepatoma cells for HCVcc permissiveness we identified new cell lines capable of supporting authentic HCVcc infection (e.g. entry, replication, protein expression and at least for Hep3B cells, *de novo *virion production). The permissiveness of PLC and Hep3B cells for HCVcc infection is clearly less robust than that achieved in the highly HCVcc-permissive Huh7 cell lines [34] and while further studies are needed to definitively and quantitatively assess the host cell factors that limit HCV infection in these cells, these two hepatoma cell lines nonetheless provide new *in vitro *tools for elucidating the cellular factors that control or mediate HCV infection, particularly those associated with HCV ISG induction or those involved in *de novo *HCVcc production and secretion. In addition, HCV sub-genomic and full-length replicons have not been previously established in Hep3B and PLC cells [33]. Thus, the clones generated in this study add to the current repertoire of sub-genomic and full-length HCV replicons already available to study specific aspects of the HCV life cycle such as HCV polyprotein processing, replication complex formation and HCV RNA replication. Expanding our arsenal of reagents and cell lines amendable for the study of HCV should certainly advance our overall understanding of the HCV viral life cycle and the cellular determinants involved, facilitating the potential development of new and more specific HCV antivirals.

## Methods

### Cells and viruses

Huh7-1 cells have been previously described [34]. The following cells were purchased from the American Type Culture Collection: PLC/PRF/5 cells (CRL-8024), HepG2 cells (HB-8065), HeLa cells (CCl-2), SW13 cells (CCl-105), and CHO-K1 cells (CCL-61). Hep3B and 293T cells were kindly provided by Dr. Lijun Rong (University of Illinois at Chicago, Chicago, IL) and Caco-2 cells by Dr. Waddah Alrefai (University of Illinois at Chicago, Chicago, IL). All human cell lines were cultured in complete Dulbecco's modified Eagle's medium (DMEM) (Hyclone, Logan, UT). CHO-K1 cells were cultured in Ham's F-12 medium (Gibco Invitrogen, Carlsbad, CA). Media was supplemented with 10% fetal bovine serum (FBS) (Hyclone), 100 units/ml penicillin, 100 mg/ml streptomycin, 2 mM L-glutamine (Gibco Invitrogen) and in the case of HCV replicon cells 500 μg/ml G418 (Invitrogen).

JFH-1 cell culture-propagated HCV (HCVcc) viral stocks were obtained by infection of naïve Huh7-1 cells at a multiplicity of infection (MOI) of 0.01 focus forming units (FFU)/cell, using medium from Huh7-1 cells electroporated with *in vitro*-transcribed full length infectious HCV JFH-1 RNA as previously described [40].

### DNA constructs

The genotype 2a infectious JFH-1 (pJFH-1), the full length (fl) JFH-1 replicon (pFLJFH) and the subgenomic (sg) JFH-1 replicon (pSGJFH) plasmids were kindly provided by Dr. Wakita (National Institute of Infectious Diseases, Tokyo, Japan) and have been previously described [12,14,38]. The human CD81 (pEE6-huCD81), human SR-BI (pZeo_hSR-BI), human CLDN1 (pZeo_CLDN1) and human OCLN (pCDNA3.1_OCLN) expression plasmids have been previously described [34].

### Pseudotyped retrovirus production and infections

Pseudotyped viruses were produced as previously described [34]. Briefly, pseudotyped viruses were generated by co-transfection of DNA vectors encoding HCV or vesicular stomatitis virus (VSV) envelope glycoproteins with an Env-deficient HIV vector carrying a luciferase reporter gene (pNL4-3-Luc-R^-^-E^-^) into 293T producer cells. The plasmids used for pseudotyped virus generation have been described previously [34]. Supernatants were collected 48 h post transfection, filtered through a 0.45 μm-pore-size filter (BD Biosciences), aliquoted, frozen and subsequently titered using the QuickTiter Lentivirus Titer Kit (Cell Biolabs, Inc., San Diego, CA) according to the manufacturer's instructions.

For infections, cells were seeded 24 h prior to infection in 96-well plates such that cell confluence at the time of infection was 90%. Triplicate wells were inoculated with equal titers of JFHpp, H77pp and VSVGpp for 6 h, washed twice with 1 × PBS following infection, and then overlaid with 200 μl cDMEM. At 72 h p.i. cultures were lysed in 20 μl of lysis reagent to measure luciferase activity (Promega, Madison, WI) using a FLUOstar Optima microplate reader (BMG Labtechnologies Inc, Durham, NC). For all pseudotype virus experiments presented herein, background-corrected (BC) HCVpp RLU values were normalized to corresponding BC-corrected VSVGpp RLU values: [(HCVpp-BC)/(VSVG-BC)].

### HCVcc infections

For infection of cell lines, each cell line was seeded 24 h prior to infection in 96-well plates such that cell confluence at the time of infection was 90%. Triplicate wells were inoculated with HCVcc at an MOI of 2.0 FFU/cell and 72 h p.i. total cellular RNA was isolated for reverse transcription followed by real-time quantitative PCR analysis (RTqPCR). For low MOI HCV RNA kinetic analysis, cells were seeded 24 h prior to infection in 6-well plates such that cell confluence at the time of infection was 80%. Triplicate wells were infected with JFH-1 HCV at an MOI of 0.01 FFU/cell. During the 7 days infection assay, infected cells were trypsinized before reaching confluence and re-plated at a 1:3 dilution to maintain active growth. At indicated times p.i., medium and/or cellular RNA was harvested from triplicate wells for titration or RTqPCR analysis, respectively.

### RNA isolation and RTqPCR

Total intracellular RNA was isolated in 1 × Nucleic Acid Purification Lysis Solution (Applied Biosystems, Foster City, CA) and purified using an ABI PRISM™ 6100 Nucleic Acid PrepStation (Applied Biosystems), as per the manufacturer's instructions. Extracellular RNA from cell supernatants or intracellular HCV RNA from lysed cell pellets was treated with 4.5 units of S7 Micrococcal nuclease (Fermentas, Glen Burnie, MD) for 30 min at room temperature, and nuclease-treated RNA was isolated by the guanidine thiocyanate (GTC) method using 1.6 × GTC containing 2 μg of murine total liver RNA and following standard protocols [68]. One μg of purified RNA was used for cDNA synthesis using the TaqMan reverse transcription reagents (Applied Biosystems), followed by SYBR green RTqPCR using an Applied Biosystems 7300 real-time thermocycler (Applied Biosystems). Thermal cycling consisted of an initial 10 min denaturation step at 95°C followed by 40 cycles of denaturation (15 s at 95°C) and annealing/extension (1 min at 60°C). HCV, human GAPDH, murine GAPDH, human CD81, human SR-BI, human CLDN1, human OCLN and human ISG56 RNA levels were determined relative to standard curves comprised of serial dilutions of plasmids containing the JFH-1 HCV cDNA or the human GAPDH, murine GAPDH, human CD81, human SR-BI, human CLDN1, human OCLN and human ISG56 coding sequences, respectively. The PCR primers used to amplify each respective amplicon were: Universal HCV primers [69] 5'-GCC TAG CCA TGG CGT TAG TA -3' (sense) and 5'- CTC CCG GGG CACTCG CAA GC-3' (anti-sense), human GAPDH [13] 5'-GAA GGT GAA GGT CGG AGT C-3' (sense) and 5'-GAA GAT GGT GAT GGG ATT TC-3' (anti-sense), murine GAPDH [70] 5'-TCT GGA AAG CTG TGG CGT G-3' (sense) and 5'-CCA GTG AGC TTC CCG TTC AG-3' (antisense), human CD81 [25] 5'-ACC TCC TGT ATC TGG AGC TGG-3' (sense) and 5'-TTG GCG ATC TGG TCC TTG TTG-3' (anti-sense), human SR-BI [25] 5'-TCG CAG GCA TTG GAC AAA CT-3' (sense) and 5'-CTC CTT ATC CTT TGA GCC CTT TT-3' (anti-sense), human CLDN1 [25] 5'- GTG GAG GAT TTA CTC CTA TGC CG-3' (sense) and 5'- ATC AAG GCA CGG GTT GCT T-3' (anti-sense), human OCLN [25] 5'- TCA AAC CGA ATC ATT ATG CAC CA-3' (sense) and 5'- AGA TGG CAA TGC ACA TCA CAA-3' (anti-sense), and human ISG56 [71] 5'- GGG CAG ACT GGC AGA AGC -3' (sense) and 5'- TAT AGC GGA AGG GAT TTG AAA GC -3' (anti-sense).

### Flow cytometric analysis of cell surface receptors

Cells were resuspended in 150 μl of FACS buffer (1 × PBS containing 2% (v/v) FBS, 0.3% (w/v) NaN_3 _and 1 mM EDTA) and incubated for 60 min at 4°C with a 1:100 dilution of antibodies specific for CD81 (AbD Serotec) or SR-BI (BD BioSciences) Following three rinses with FACS buffer, bound antibodies were detected by incubation for 1 h at 4°C with an phycoerythrin (PE)-conjugated anti-mouse (BD Pharmingen) antibody at a dilution of 1:200. Cells stained with an irrelevant mouse immunoglobulin G (IgG) antibody and respective PE-conjugated secondary antibody served as negative controls. Cells were washed three times, fixed in FACS buffer containing 4% (w/v) PFA, and analyzed by flow cytometry using the DakoCytomation CyAn system (Dako, Carpinteria, CA) and Summit Software v4.3 (Dako).

### Indirect immunofluorescence analysis

Cells were fixed with 4% PFA (Sigma, St. Louis, MO) at indicated times and immunofluorescence analysis was performed as previously described [36]. Briefly, fixed cultures were rinsed three times with 1 × PBS, permeabilized with 50% Methanol/50% acetone (v/v) (Fisher) and subsequently blocked for 1 h with 1 × PBS containing 3% (w/v) bovine serum albumin (BSA) (Sigma) and 10% (v/v) FBS. Cells were stained with a 1:750 dilution of a mouse anti-human CLDN1 (Abnova, Taipei, Taiwan) or mouse anti-human OCLN (Zymed, San Francisco, CA) primary antibody overnight at 4°C, followed by incubation with a 1:750 dilution of an anti-mouse Alexa-555 conjugated secondary antibody (Molecular Probes) for 1 h at room temperature. Cell nuclei were stained by Hoechst dye. Bound antibodies were visualized via confocal microscopy (630X, Zeiss LSM 510, Germany) and compared to negative control samples stained with an irrelevant mouse control antibody (Santa Cruz Biotechnology) and Alexa-555 conjugated secondary antibody. Images were analyzed using Zeiss LSM Alpha Imager Browser v4.0 software (Zeiss), and brightness and contrast were adjusted using Adobe^®^Photoshop^® ^(San Jose, CA).

### HCV RNA transfection and G418-resistant colony formation

HCV sg and fl 2a replicons were established as previously described [37]. Briefly, 1 μg of *in vitro*-transcribed HCV genotype sg2a or fl2a replicon RNA was transfected into cells using a modified electroporation protocol [72]. Transfected cells were diluted 1:500, seeded in 100 mm tissue culture dishes and maintained in the presence of G418 (Invitrogen) at a concentration of 500 μg/ml until all cells died or distinct G418-resistant cell colonies formed. To visualize colony formation, cells were fixed with ethanol and stained with crystal violet. Alternatively, colonies were expanded to establish replicon cell lines. HCVcc^*G418 *^was harvested from stable fl 2a Huh7 replicon cells and infectivity titers were determined as described below.

### Infectivity titration assay and immuncytochemical staining of HCV E2 protein

Culture supernatants were serially diluted 10-fold and used to infect 96-well Huh7 cultures. At 24 h p.i., cultures were overlayed with complete DMEM containing a final concentration of 0.25% methylcellulose (Fluka BioChemika, Switzerland). Seventy-two hours p.i., cells were fixed in 4% paraformaldehyde (Sigma), and immunohistochemically stained for HCV E2 using a human monoclonal anti-E2 antibody C1 [39] (a gift from Drs. Mansun Law and Dennis Burton, The Scripps Research Institute) as described [40]. Viral titers are expressed as FFU/ml, determined by the average E2-positive foci number detected at the highest HCV-positive dilution.

### Quantification of cell-associated infectious HCVcc

To quantify intracellular HCVcc, HCV-infected cell cultures were washed three times with 1 × PBS, trypsinized for 5 min at room temperature and cells resuspended in 1 ml of 10% cDMEM. Cells were pelleted at 1,400 RPM for 5 min at 4°C, frozen and then thawed three times using a 95% ethanol:dry ice bath. Samples were then centrifuged at 1,400 RPM for 5 min at 4°C to remove cell debris. The supernatant fractions were used for titration of infectious HCVcc or for HCV RNA analysis, as described above.

### Statistics

Data are presented as the means ± standard deviation (SD). Significant differences were determined by one-way analysis of variance (ANOVA) followed by Tukey's post hoc *t *test (GraphPad Prism^© ^Software).

## Abbreviations

HCV: Hepatitis C virus; HCC: Hepatocellular carcinoma; JFH-1: Japanese Fulminant Hepatitis; HCVcc: Hepatitis C virus cell-cultured produced; HCVpp: HCV pseudotype particles; VSVGpp: Vesicular stomatitis virus G protein pseudotype particles; sg: Subgenomic; fl: Full-length; MOI: Multiplicity of infection; FFU: Focus forming units; RTqPCR: Real-time quantitative PCR; SR-BI: Scavenger receptor class B member I; CLDN1: Claudin-1; OCLN: Occludin; p.i.: Post infection; ISG: Interferon stimulated gene.

## Competing interests

The authors declare that they have no competing interests.

## Authors' contributions

BS and SLU designed the study and drafted the manuscript. BS, NB, XY and PC performed the experiments and participated in the data analysis. All authors read and approved the final manuscript.

## Supplementary Material

Additional file 1 **Figure S1**. Establishment of HepG2-CD81 cells. (A) HepG2 cells, seeded in a 100 mm tissue culture dish, were transfected with a vector control (left panel) or pEE6-huCD81 (right panel) using Lipofectamine2000™ according to the manufacturer's instructions. Twenty-four hours post transfection, cell culture medium was supplemented with G418 at 500 μg/ml. Approximately 3 weeks post transfection, G418-resistant colonies were trypsinized, pooled and cell surface expression of CD81 was determined by flow cytometric analysis using a mouse anti-CD81 antibody and an anti-mouse secondary antibody conjugated with PE. (B) Cells expressing mean CD81 values of greater than 10^2 ^were sorted using a Becton Dickinson MoFlo cell sorter, expanded and re-analyzed for cell surface CD81 expression by flow cytometry as described above (Sort #1). (C) Cells expressing mean CD81 values of greater than 10^2 ^were sorted again, expanded and reanalyzed for cell surface CD81 expression by flow cytometry as described above. (Sort #2) Cells obtained after two rounds of cell sorting were aliquoted, frozen and designated HepG2-CD81 cells. Shaded regions represent cells stained with a monoclonal mouse control primary antibody and respective anti-mouse PE-conjugated secondary antibody. Additional file 1 Figure S2. Morphological analysis of human hepatoma cell lines. (A) Huh7, (B) HepG2-CD81, (C) Hep3B and (D) PLC cells were plated at 5 × 10^4 ^cells/well in a 12-well plate and photographed 2 days after plating (magnification, ×100). Additional file 1 Figure S3. HCVpp infection of different Huh7 cell lines. Huh7 cells lines from different laboratories [34] were infected with equal amounts of JFHpp, H77pp or VSVGpp. HCVpp entry is expressed as relative light units (RLU) ± sem for triplicate samples determined 72 h p.i. Additional file 1 Figure S4. HCVcc infection of IFN-cured fl2a replicon clones. PLC and Hep3B fl2a replicon cell lines replicating HCV RNA at levels ≥ 4.4 × 10^6 ^copies/μg RNA were cured of HCV by co-treatment with 100 U/ml each of IFN-β and IFN-γ for 3 weeks. The absence of HCV RNA was confirmed by RTqPCR analysis. Parental Huh7, PLC and Hep3B cells and cured fl2a replicon clones were then infected with HCVcc at an MOI of 2.0 FFU/cell. Intracellular RNA was collected 48 and 72 h p.i. and HCV RNA was quantified by RTqPCR, normalized to GAPDH and is displayed as HCV RNA copies/μg total cellular RNA (means ± SD for triplicate samples). wt = wild-type parental cells, C# = clone number. Additional file 1 Figure S5. HCV RNA replication in Huh7, PLC, Hep3B and HepG2-CD81 cells transfected with *in vitro*-transcribed full-length infectious HCV JFH-1 RNA. Huh7, PLC, Hep3B and HepG2-CD81 cells were transfected with 7 μg of *in vitro*-transcribed full-length infectious JFH-1 RNA or a replication-deficient GND mutant via electroporation. HCV RNA was quantified by RTqPCR on indicated days post-transfection, normalized to GAPDH and is displayed as mean HCV RNA copies/μg total cellular RNA ± SD. Click here for file
