# Supplementary material for: Permissiveness of human hepatoma cell lines for HCV infection
Source: Virol J. 2012 Jan 24;9:30. doi: 10.1186/1743-422X-9-30 (PMC3317838; doi:10.1186/1743-422X-9-30)
Supplement: Additional file 1 — Figure S1. Establishment of HepG2-CD81 cells. (A) HepG2 cells, seeded in a 100 mm tissue culture dish, were transfected with a vector control (left panel) or pEE6-huCD81 (right panel) using Lipofectamine2000™ according to the manufacturer's instructions. Twenty-four hours post transfection, cell culture medium was supplemented with G418 at 500 μg/ml. Approximately 3 weeks post transfection, G418-resistant colonies were trypsinized, pooled and cell surface expression of CD81 was determined by flow cytometric analysis using a mouse anti-CD81 antibody and an anti-mouse secondary antibody conjugated with PE. (B) Cells expressing mean CD81 values of greater than 102 were sorted using a Becton Dickinson MoFlo cell sorter, expanded and re-analyzed for cell surface CD81 expression by flow cytometry as described above (Sort #1). (C) Cells expressing mean CD81 values of greater than 102 were sorted again, expanded and reanalyzed for cell surface CD81 expression by flow cytometry as described above. (Sort #2) Cells obtained after two rounds of cell sorting were aliquoted, frozen and designated HepG2-CD81 cells. Shaded regions represent cells stained with a monoclonal mouse control primary antibody and respective anti-mouse PE-conjugated secondary antibody. Additional file 1 Figure S2. Morphological analysis of human hepatoma cell lines. (A) Huh7, (B) HepG2-CD81, (C) Hep3B and (D) PLC cells were plated at 5 × 10^4^ cells/well in a 12-well plate and photographed 2 days after plating (magnification, ×100). Additional file 1 Figure S3. HCVpp infection of different Huh7 cell lines. Huh7 cells lines from different laboratories [34] were infected with equal amounts of JFHpp, H77pp or VSVGpp. HCVpp entry is expressed as relative light units (RLU) ± sem for triplicate samples determined 72 h p.i. Additional file 1 Figure S4. HCVcc infection of IFN-cured fl2a replicon clones. PLC and Hep3B fl2a replicon cell lines replicating HCV RNA at levels ≥ 4.4 × 10^6^ copies/μg RNA were cured of HCV b [file 1743-422X-9-30-S1.PDF]

Sainz *et al.*, Supplementary Figure S1

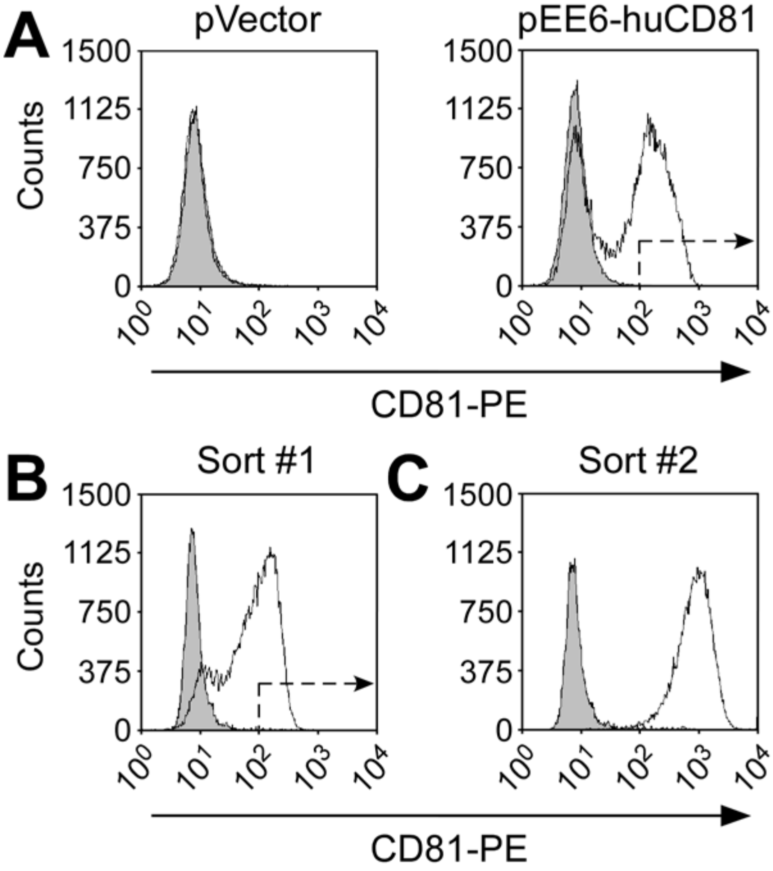

Supplementary Figure S1. Establishment of HepG2-CD81 cells. (A) HepG2 cells, seeded in a 100mm tissue culture dish, were transfected with a vector control (left panel) or pEE6-huCD81 (right panel) using Lipofectamine2000™ according to the manufacturer's instructions. Twenty-four hours post transfection, cell culture medium was supplemented with G418 at 500 μg/ml. Approximately three weeks post transfection, G418-resistant colonies were trypsinized, pooled and cell surface expression of CD81 was determined by flow cytometric analysis using a mouse anti-CD81 antibody and an anti-mouse secondary antibody conjugated with PE. (B) Cells expressing mean CD81 values of greater than 10^2 were sorted using a Becton Dickinson MoFlo cell sorter, expanded and re-analyzed for cell surface CD81 expression by flow cytometry as described above (Sort #1). (C) Cells expressing mean CD81 values of greater than 10^2 were sorted again, expanded and reanalyzed for cell surface CD81 expression by flow cytometry as described above. (Sort #2) Cells obtained after two rounds of cell sorting were aliquoted, frozen and designated HepG2-CD81 cells. Shaded regions represent cells stained with a monoclonal mouse control primary antibody and respective anti-mouse PE-conjugated secondary antibody.

Sainz *et al.*, Supplementary Figure S2

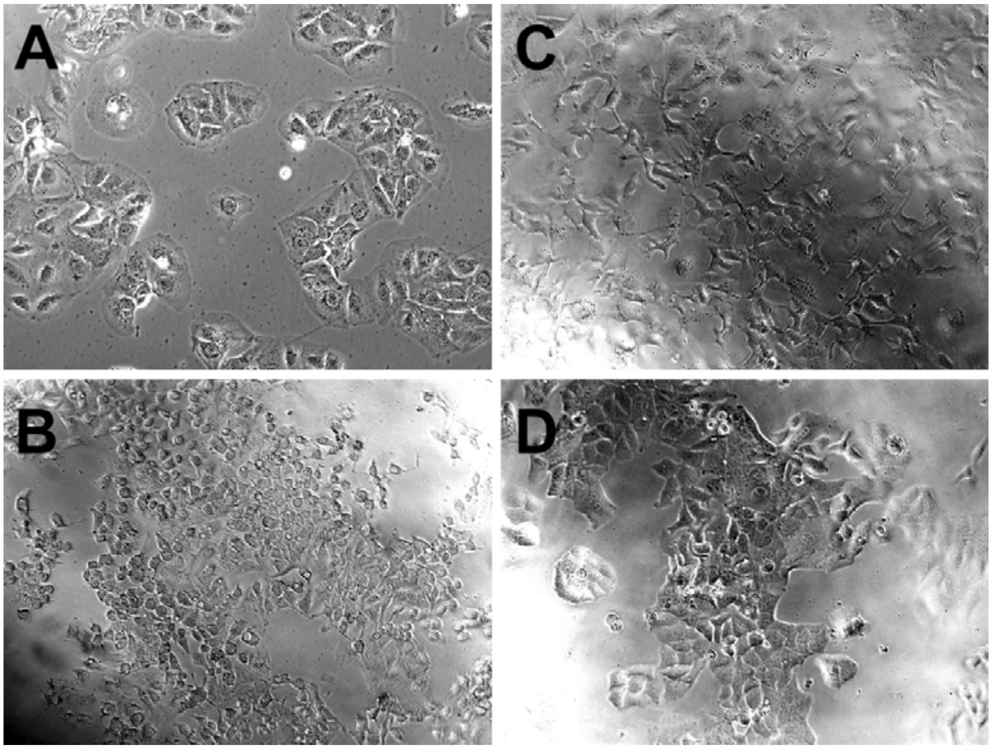

Supplementary Figure S2. Morphological analysis of human hepatoma cell lines. (A) Huh7, (B) HepG2-CD81, (C) Hep3B and (D) PLC cells were plated at 5×10^4 cells/well in a 12-well plate and photographed 2 days after plating (magnification, x100).

Sainz *et al.*, Supplementary Figure S3

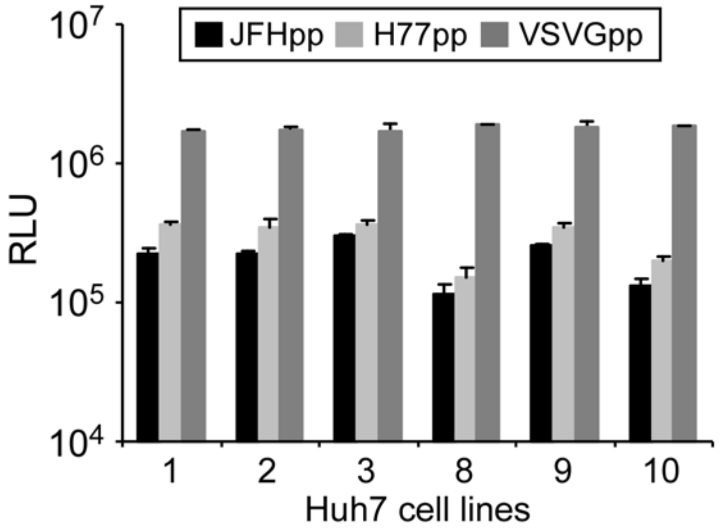

Supplementary Figure S3. HCVpp infection of different Huh7 cell lines. Huh7 cells lines from different laboratories [33] were infected with equal amounts of JFHpp, H77pp or VSVGpp. HCVpp entry is expressed as relative light units (RLU) ± sem for triplicate samples determined 72 h p.i.

Sainz *et al.*, Supplementary Figure S4

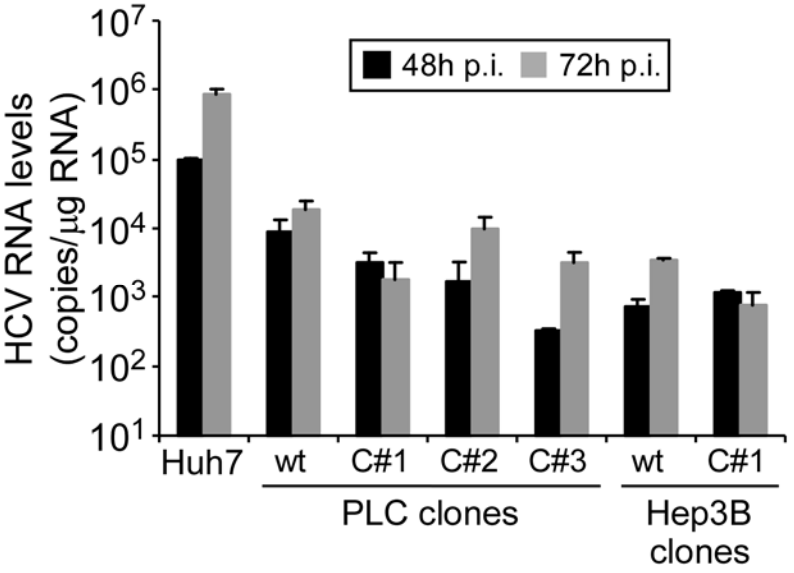

Supplementary Figure S4. HCVcc infection of IFN-cured fl2a replicon clones. PLC and Hep3B fl2a replicon cell lines replicating HCV RNA at levels ≥ 4.4×10^6 copies/μg RNA were cured of HCV by co-treatment with 100 U/ml each of IFN-β and IFN-γ for three weeks. The absence of HCV RNA was confirmed by RTqPCR analysis. Parental Huh7, PLC and Hep3B cells and cured fl2a replicon clones were then infected with HCVcc at an MOI of 2.0 FFU/cell. Intracellular RNA was collected 48 and 72 h p.i. and HCV RNA was quantified by RTqPCR, normalized to GAPDH and is displayed as HCV RNA copies/μg total cellular RNA (means ± SD for triplicate samples). wt = wild-type parental cells, C# = clone number.

Sainz *et al.*, Supplementary Figure S5

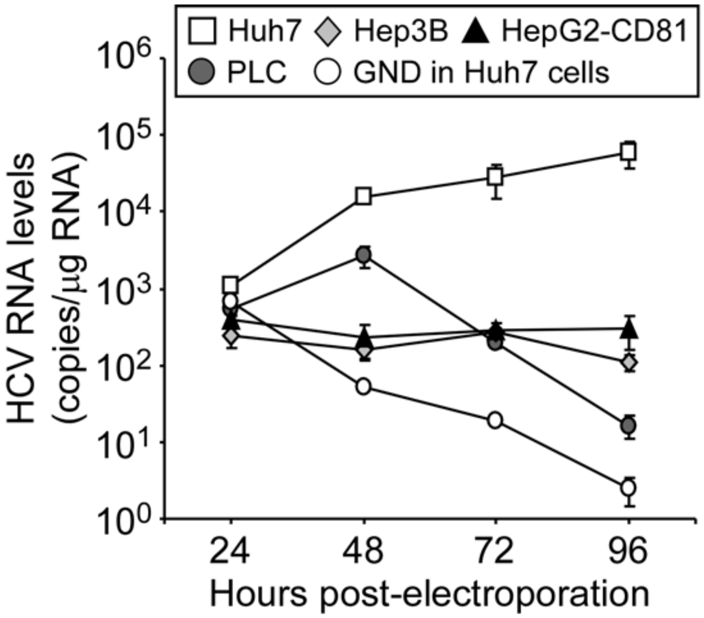

Supplementary Figure S5. HCV RNA replication in Huh7, PLC, Hep3B and HepG2-CD81 cells transfected with in vitro-transcribed full-length infectious HCV JFH-1 RNA. Huh7, PLC, Hep3B and HepG2-CD81 cells were transfected with 7 μg of in vitro-transcribed full-length infectious JFH-1 RNA or a replication-deficient GND mutant via electroporation. HCV RNA was quantified by RTqPCR on indicated days post-transfection, normalized to GAPDH and is displayed as mean HCV RNA copies/μg total cellular RNA ± SD.
